# Supplementary material for: SwarmTCR: a computational approach to predict the specificity of T cell receptors
Source: BMC Bioinformatics. 2021 Sep 7;22:422. doi: 10.1186/s12859-021-04335-w (PMC8422754; doi:10.1186/s12859-021-04335-w)
Supplement: Supplementary file 1 — Additional file 1. Supplementary Figures and Tables. [file 12859_2021_4335_MOESM1_ESM.pdf]

SwarmTCR: a computational approach to predict the specificity of T Cell Receptors

Supplementary Material

Ryan Ehrlich[1,], Larisa Kamga[2], Anna Gil[3], Katherine Luzuriaga[2], LiisaSelin[3], and Dario Ghersi[1,*]

[1]School of Interdisciplinary Informatics, University of Nebraska at Omaha, Omaha, NE 68182, USA
[2]Program in Molecular Medicine, University of Massachusetts Medical School, Worcester, MA 01655, USA and
[3]Department of Pathology, University of Massachusetts Medical School, Worcester, MA 01655, USA.
* To whom correspondence should be addressed

BS data		CDR1 Weight	CDR2 Weight	CDR2.5 Weight	CDR3 Weight	SwarmTCR AP	TCRdist AP	p-value
GLC α, β:	Mean	0.153, 0.109	0.071, 0.113	0.094, 0.014	0.682, 0.764	0.75, 0.771	0.728, 0.662	1.123e^{-7}, 1.166e^{-50}
	SD	0.08, 084	0.05, 0.074	0.069, 0.041	0.019, 0.032	0.02, 0.016	0.018, 0.02	
YVL α, β:	Mean	0.012, 0.174	0.115, 0.048	0.084, 0.012	0.749, 0.766	0.717, 0.846	0.681, 0.74	5.452e^{-18}, 2.030e^{-84}
	SD	0.031, 0.068	0.075, 0.044	0.086, 0.037	0.032, 0.024	0.017, 0.008	0.016, 0.008	
GIL α, β:	Mean	0.113, 0.151	0.057, 0.182	0.164, 0.025	0.666, 0.641	0.708, 0.687	0.682, 0.654	3.236e^{-4}, 9.877e^{-06}
	SD	0.097, 0.046	0.061, 0.059	0.097, 0.05	0.026, 0.021	0.33, 0.36	0.37, 0.35	
SC data		CDR1 Weight	CDR2 Weight	CDR2.5 Weight	CDR3 Weight	SwarmTCR AP	TCRdist AP	p-value
GLC α, β:	Mean	0.178, 0.039	0.044, 0.153	0.118, 0.128	0.141, 0.199	0.797	0.774	0.035
	SD	0.063, 0.074	0.061, 0.082	0.079, 0.09	0.051, 0.056	0.052	0.054	
YVL α, β:	Mean	0.178, 0.069	0.087, 0.091	0.181, 0.031	0.226, 0.137	0.691	0.643	0.002
	SD	0.082, 0.084	0.095, 0.086	0.082, 0.058	0.043, 0.077	0.076	0.077	
GIL α, β:	Mean	0.140, 0.121	0.057, 0.156	0.093, 0.145	0.095, 0.191	0.805	0.793	0.062
	SD	0.098, 0.094	0.088, 0.074	0.093, 0.088	0.077, 0.058	0.031	0.032	
LLW α, β:	Mean	0.201, 0.03	0.044, 0.081	0.151, 0.07	0.257, 0.165	0.778	0.712	0.002
	SD	0.109, 0.053	0.085, 0.096	0.129, 0.093	0.106, 0.099	0.058	0.135	
NLV α, β:	Mean	0.189, 0.097	0.021, 0.137	0.053, 0.044	0.27, 0.189	0.452	0.446	0.683
	SD	0.087, 0.117	0.047, 0.099	0.088, 0.078	0.098, 0.112	0.081	0.081	

Table 1: The mean CDR loop weights and precision-recall results for BS & SC data is showed above. As specified in the first column, the two values in columns 2-5 corresponBS to the α and β chain, respectively. Columns 6 and 7 display AP results for SwarmTCR and TCRdist and the t-test statistic for performance comparison is in the final columnn. Weights were collected from every optimized set of the 50 iterations performed (LLW = LLWNGPMAV, NLV = NLVPMVATV).

SC GLCTLVAML data		CDR1 Weight	CDR2 Weight	CDR2.5 Weight	CDR3 Weight	SwarmTCR AP	TCRdist AP	p-value
Confidence 0 α, β:	Mean	0.187, 0.039	0.02, 0.176	0.139, 0.1	0.139, 0.2	0.813	0.783	0.008
	SD	0.059, 0.068	0.044, 0.066	0.072, 0.079	0.056, 0.05	0.053	0.056	
Confidence 2 α, β:	Mean	0.19, 0.057	0.036, 0.163	0.121, 0.103	0.124, 0.206	0.811	0.791	0.084
	SD	0.066, 0.089	0.067, 0.094	0.081, 0.092	0.054, 0.051	0.056	0.06	
Confidence 3 α, β:	Mean	0.197, 0.047	0.032, 0.172	0.128, 0.094	0.14, 0.19	0.818	0.79	0.021
	SD	0.051, 0.076	0.057, 0.072	0.085, 0.074	0.06, 0.049	0.057	0.061	
Shuffled CDRs α, β:	Mean	0.178, 0.049	0.219, 0.054	0.179, 0.087	0.179, 0.054	0.473	0.451	0.088
	SD	0.088, 0.06	0.105, 0.077	0.103, 0.094	0.109, 0.083	0.069	0.06	

SC GILGFVFTL data		CDR1 Weight	CDR2 Weight	CDR2.5 Weight	CDR3 Weight	SwarmTCR AP	TCRdist AP	p-value
Confidence 0 α, β:	Mean	0.177, 0.138	0.044, 0.149	0.054, 0.173	0.074, 0.19	0.805	0.792	0.117
	SD	0.098, 0.113	0.076, 0.098	0.082, 0.085	0.066, 0.079	0.043	0.039	
Confidence 2 α, β:	Mean	0.147, 0.134	0.044, 0.141	0.081, 0.172	0.09, 0.191	0.814	0.805	0.196
	SD	0.091, 0.093	0.069, 0.081	0.095, 0.078	0.074, 0.069	0.029	0.034	
Confidence 3 α, β:	Mean	0.152, 0.117	0.031, 0.185	0.057, 0.165	0.094, 0.198	0.81	0.799	0.121
	SD	0.11, 0.113	0.061, 0.087	0.079, 0.092	0.079, 0.087	0.034	0.033	
Shuffled CDRs α, β:	Mean	0.036, 0.217	0.045, 0.235	0.021, 0.214	0.008, 0.224	0.779	0.685	$2.376e^{-21}$
	SD	0.052, 0.029	0.05, 0.036	0.043, 0.043	0.018, 0.031	0.034	0.042	

SC YVLDHLIVV data		CDR1 Weight	CDR2 Weight	CDR2.5 Weight	CDR3 Weight	SwarmTCR AP	TCRdist AP	p-value
Confidence 0 α, β:	Mean	0.195, 0.079	0.072, 0.105	0.158, 0.025	0.228, 0.137	0.708	0.67	0.011
	SD	0.081, 0.084	0.087, 0.087	0.1, 0.054	0.06, 0.081	0.075	0.069	
Confidence 2 α, β:	Mean	0.181, 0.081	0.095, 0.093	0.152, 0.033	0.223, 0.143	0.711	0.664	0.002
	SD	0.089, 0.086	0.085, 0.081	0.1, 0.064	0.05, 0.078	0.078	0.074	
Confidence 3 α, β:	Mean	0.19, 0.056	0.099, 0.09	0.166, 0.057	0.215, 0.129	0.688	0.641	0.005
	SD	0.084, 0.072	0.086, 0.082	0.086, 0.075	0.055, 0.057	0.077	0.084	
Shuffled CDRs α, β:	Mean	0.168, 0.039	0.189, 0.035	0.237, 0.017	0.267, 0.048	0.356	0.239	$4.264e^{-10}$
	SD	0.118, 0.061	0.132, 0.069	0.117, 0.042	0.136, 0.076	0.091	0.075	

SC LLWNGPMAV data	CDR1 Weight	CDR2 Weight	CDR2.5 Weight	CDR3 Weight	SwarmTCR AP	TCRdist AP	p-value
Confidence 0 α, β: Mean	0.182, 0.042	0.042, 0.077	0.168, 0.063	0.273, 0.153	0.777	0.719	0.004
SD	0.136, 0.081	0.096, 0.084	0.148, 0.088	0.098, 0.103	0.061	0.123	
Confidence 2 α, β: Mean	0.167, 0.03	0.06, 0.076	0.17, 0.077	0.261, 0.159	0.793	0.716	0.001
SD	0.132, 0.065	0.112, 0.097	0.152, 0.1	0.098, 0.105	0.056	0.14	
Confidence 3 α, β: Mean	0.186, 0.052	0.045, 0.088	0.162, 0.064	0.225, 0.179	0.781	0.742	0.044
SD	0.11, 0.078	0.087, 0.097	0.12, 0.093	0.098, 0.111	0.06	0.12	
Shuffled CDRs α, β: Mean	0.244, 0.045	0.209, 0.027	0.223, 0.008	0.237, 0.007	0.65	0.485	$4.131e^{-22}$
SD	0.031, 0.056	0.054, 0.039	0.041, 0.016	0.04, 0.015	0.064	0.066	

SC NLVPMVATV data	CDR1 Weight	CDR2 Weight	CDR2.5 Weight	CDR3 Weight	SwarmTCR AP	TCRdist AP	p-value
Confidence 0 α, β: Mean	0.232, 0.078	0.035, 0.152	0.052, 0.037	0.248, 0.167	0.423	0.426	0.892
SD	0.09, 0.109	0.079, 0.102	0.091, 0.073	0.091, 0.137	0.086	0.077	
Confidence 2 α, β: Mean	0.194, 0.066	0.017, 0.18	0.054, 0.056	0.236, 0.197	0.406	0.404	0.885
SD	0.092, 0.103	0.049, 0.116	0.096, 0.079	0.097, 0.139	0.082	0.083	
Confidence 3 α, β: Mean	0.214, 0.034	0.035, 0.191	0.054, 0.05	0.228, 0.194	0.404	0.403	0.921
SD	0.09, 0.07	0.074, 0.103	0.09, 0.077	0.08, 0.113	0.084	0.085	
Shuffled CDRs α, β: Mean	0.218, 0.145	0.173, 0.011	0.115, 0.127	0.113, 0.098	0.173	0.163	0.223
SD	0.131, 0.116	0.106, 0.037	0.139, 0.105	0.084, 0.109	0.037	0.041	

Table 2: The mean CDR loop weights and precision-recall results for SC data is showed above. As specified in the first column, the two values in columns 2-5 correspond to the α and β chain, respectively. Columns 6 and 7 display AP results for SwarmTCR and TCRdist and the t-test statistic for performance comparison is in the final column. Weights were collected from every optimized set of the 50 iterations performed (LLW = LLWNGPMAV, NLV = NLVPMVATV).Confidence 0, 2, and 3 refer to VDJDB quality scores, see methods. Shuffled CDRs indicate random placement of CDR loops, see methods.

BS data	SwarmTCR AUROC	TCRdist AUROC
GLC α: Mean	0.897	0.892
SD	0.007	0.007
GLC β: Mean	0.916	0.882
SD	0.005	0.005
YVL α: Mean	0.834	0.822
SD	0.010	0.011
YVL β: Mean	0.897	0.846
SD	0.005	0.005
GIL α: Mean	0.925	0.921
SD	0.008	0.008
GIL β: Mean	0.942	0.933
SD	0.007	0.007
SC data	SwarmTCR AUROC	TCRdist AUROC
GLC $\alpha\beta$: Mean	0.897	0.887
SD	0.0.32	0.0.35
YVL $\alpha\beta$: Mean	0.860	0.845
SD	0.054	0.049
GIL $\alpha\beta$: Mean	0.880	0.881
SD	0.021	0.020
LLW $\alpha\beta$: Mean	0.888	0.884
SD	0.040	0.038
NLV $\alpha\beta$: Mean	0.690	0.684
SD	0.057	0.049

Table 3: An extension of the previous table, these AUROC scores and standard deviations show each repertoire tested (BS and SC).

Virus	Peptide Gene	Peptide	HLA Type	SC TCR count	BS α chain count	BS β chain count
Cancer/testis antigen 1	NY-ESO-1	APRGPHGGAASGL	HLA-B*07:02	2	0	0
Cancer/testis antigen	CTAG1B	SLLMWITQC	HLA-A*02:01	2	2	2
CMV	pp65	CPSQEPMSIYVY	HLA-B*35:08	0	4	2
CMV	IE1	CVETMCNEY	HLA-B*18	0	0	2
CMV	IE1	DEEDAIAAY	HLA-B18	0	0	2
CMV	IE1	ELRRKMMYM	HLA-B*08	2	0	4
CMV	pp65	FPTKDVAL	HLA-B*35:08	0	0	10
CMV	pp65	IPSINVHHY	HLA-B*35:01	0	0	28
CMV	UL83	NLVPMVATV	HLA-A*02:01	186	75	246
CMV	IE1	QIKVRVDMV	HLA-B*08	7	2	7
CMV	IE1	QIKVRVKMV	HLA-B*08	0	0	11
CMV	pp65	RPHERNGFTVL	HLA-B*07:02	0	0	21
CMV	pp65	TPRVTGGGAM	HLA-B*07	7	6	104
CMV	IE1	VLEETSVML	HLA-A*02:01	0	0	10
CMV	pp50	VTEHDTLLY	HLA-A*01:01	0	0	10
CMV	pp65	YSEHPTFTSQY	HLA-A*01:01	0	0	20
DENV1	NS3	GTSGSPIVNR	HLA-A*11:01	3	3	62
DENV2	NS3	GTSGSPIIDK	HLA-A*11:01	0	0	19
DENV2	NS3	GTSGSPIVDR	HLA-A*11:01	2	0	0
DENV3/4	NS3	GTSGSPIINR	HLA-A*11:01	3	3	48
EBV	EBNA4	AVFDRKSDAK	HLA-A*11:01	0	0	10
EBV	BZLF1	EPLPQGQLTAY	HLA-B*35:01	5	13	29
EBV	EBNA3	FLRGRAYGL	HLA-B*35:01	10	12	17
EBV	BMLF1	GLCTLVAML	HLA-A*02:01	194	7448	7512
EBV	EBNA1	HPVGEADYFEY	HLA-B*35:01	5	24	24
EBV	EBNA4	IVTDFSVIK	HLA-A*11:01	5	0	23
EBV	BZLF1	LPEPLPQGQLTAY	HLA-B*35:08:01	7	4	5
EBV	BZLF1	RAKFKQLL	HLA-B*08:01	0	0	156
EBV	EBNA3A	RPPIFIRRL	HLA-B*07:02	0	0	27
EBV	EBNA3B	VSFIEFVGW	HLA-B*57:01	0	0	14
EBV	EBNA3A	YPLHEQHGM	HLA-B*35:01	0	0	9
EBV	BRLF1	YVLDHLIVV	HLA-A*02:01	123	9325	13246
HCV	NS5B	ARMILMTHF	HLA-B*27	0	0	16
HCV	NS3	ATDALMTGY	HLA-A*01	0	0	139
HCV	NS3	CINGVCWTV	HLA-A*02	21	21	39
HCV	POLG	HSKKKCDEL	HLA-B*08:01:29	0	0	31
HCV	NS3	KLVALGINAV	HLA-A*02	37	51	66
HIV-1	p24	EIYKRWII	HLA-B*08	0	0	20
HIV-1	Nef	FLKEKGGL	HLA-B*08	4	4	43
HIV-1	Nef	FLKEMGGL	HLA-B*08	3	3	4
HIV-1	Nef	FLKEQGGL	HLA-B*08	3	3	4
HIV-1	Nef	FLKETGGL	HLA-B*08	3	3	4
HIV-1	Vpr	FPRPWLHGL	HLA-B*08	0	0	30
HIV-1	p24	GLNKIVRMY	HLA-B*15	0	0	13
HIV-1	p24	GPGHKARVL	HLA-B*07:02	0	0	56
HIV-1	p24	GPGMKARVL	HLA-B*07:02	0	0	4
HIV-1	Vif	HPKVSSEVHI	HLA-B*42:01	0	0	25
HIV-1	Int	IIKDYGKQM	HLA-B*42:01	0	0	18
HIV-1	Pol	ILKEPVHGV	HLA-A*02	0	0	7
HIV-1	p24	ISPRTLNAW	HLA-B*57	0	0	22
HIV-1	p24	KAFSPEVIPMF	HLA-B*57	0	20	111
HIV-1	p24	KINAWIKVV	HLA-A*02:01	0	0	3
HIV-1	p24	KRWIILGLNK	HLA-B*27:05	0	6	124
HIV-1	p24	KRWIIMGLNK	HLA-B*27:05	0	11	31
HIV-1	Int	LPPIVAKEI	HLA-B*42:01	0	0	20
HIV-1	p24	QASQEVKNW	HLA-B*57:01	0	0	8
HIV-1	Nef	QVPLRPMTYK	HLA-A*03:01	0	8	33
HIV-1	p24	RLRPGGKKK	HLA-A*03:01	0	0	9
HIV-1	p17	SLYNTVATL	HLA-A*02:01	0	0	23
HIV-1	p24	TAFTIPSI	HLA-B*51:193	12	12	13

Continued on next page

Supplementary Table 4 – continued from previous page						
Virus	Peptide Gene	Peptide	HLA Type	SC TCR count	BS α chain count	BS β chain count
HIV-1	p24	TLNAWVKVV	HLA-A*02:01	0	0	4
HIV-1	Nef	TPGPGVRYPL	HLA-B*42:01	0	0	20
HIV-1	Nef	TQGYFPDWQNY	HLA-B*15	0	0	2
Homo sapeins	BST2	LLLGIGILV	HLA-A*02	0	12	9
Homo sapiens	n/a	Naïve	n/a	44	0	0
Homo sapiens	IMP2	NLSALGIFST	HLA-A*02	0	0	18
Homo sapiens	WT1	RMFPNAPYL	HLA-A*02	2	2	2
Melanoma	MLANA	AAGIGILTV	HLA-A*02:01:48	4	2	2
Melanoma	PMEL17	ALEPGPVTA	HLA-A*02:01	2	0	0
Melanoma	MLANA	ELAGIGILTV	HLA-A*02:01:48	6	5	11
Melanoma	PMEL17	YLEAGPVTA	HLA-A*02:01	2	0	0
Melanoma	PMEL17	YLEPGAVTA	HLA-A*02:01	3	0	0
Melanoma	PMEL17	YLEPGPATA	HLA-A*02:01	2	0	0
Melanoma	PMEL17	YLEPGPVAA	HLA-A*02:01	2	0	0
Melanoma	PMEL17	YLEPGPVTA	HLA-A*02:01	2	0	0
Melanoma	PMEL17	YLEPGPVTV	HLA-A*02:01	3	0	0
HSV-2	VP22	RPRGEVRFL	HLA-B*07:02	42	32	29
HTLV-1	TAX	LLFGYPVYV	HLA-A*02:01:48	3	3	3
Influenza A	NP177	LPRRSGAAGA	HLA-B*07:02	0	0	7
Influenza A	NP44	CTELKLSDY	HLA-A*01:01	0	0	4
Influenza A	M1	GILEFVFTL	HLA-A*02:01	2	0	0
Influenza A	M1	GILGFVFTL	HLA-A*02:01	402	3881	2964
InfluenzaA	NA-231	CVNGSCFTV	HLA-A*02:01	12	12	14
Yellow Fever Virus	NS4B	LLWNGPMAV	HLA-A*02:01	202	192	198

Table 4: The number of TCRs or chains (columns 5-7) in our dataset that are specific for each peptide (column 3). Peptide species, peptide gene, and HLA type can be seen in columns 1, 2, and 3, respectively.

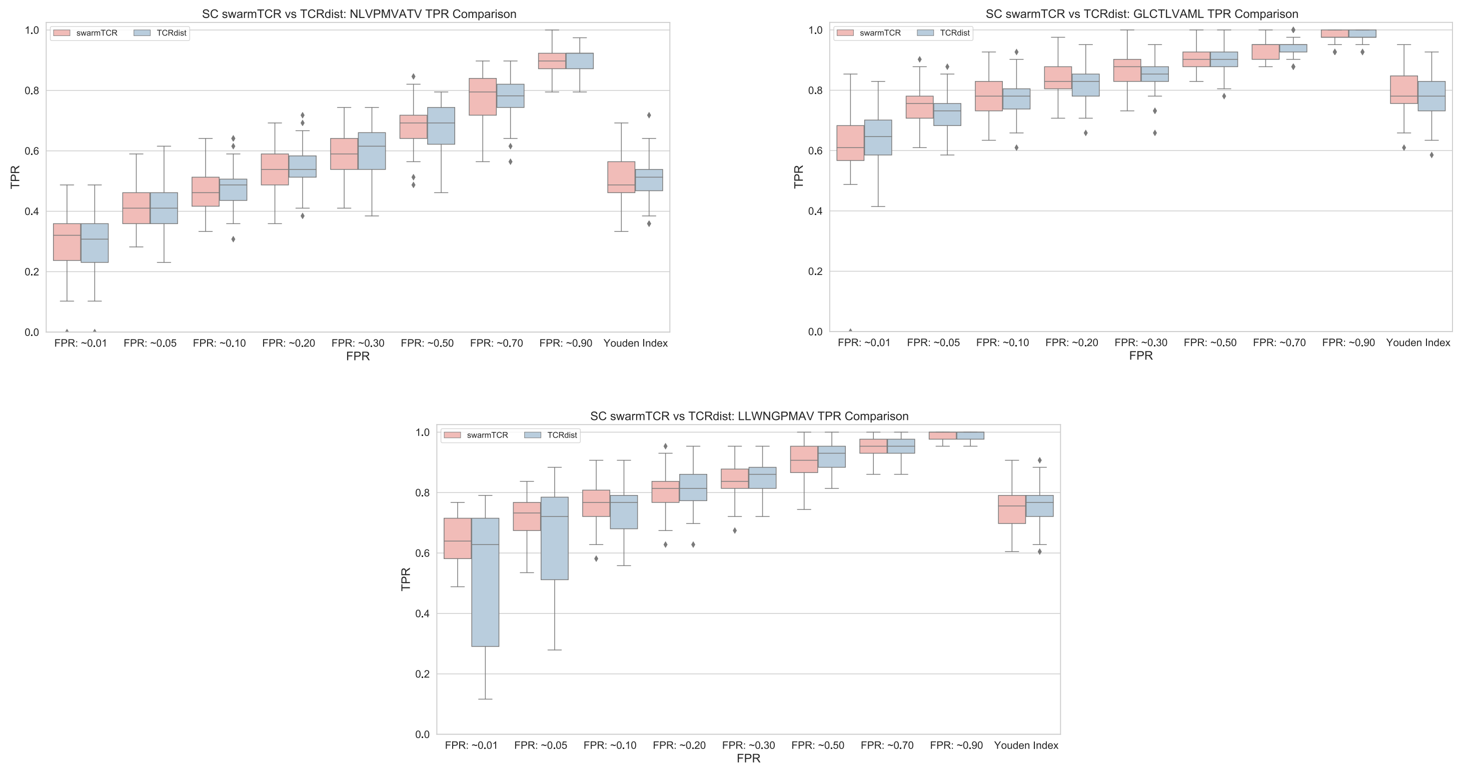

Figure 1: FPRs and their associated TPRs are plotted at thresholds 0.01, 0.1, 0.2, 0.3, 0.5, 0.7, and 0.9 for both swarmTCR and TCRdist (see legend). These plots show the results of the following SC repertoires, top to bottom, left to right: NLVPMVATV, GLCTLVAML, LLWNGPMAV.

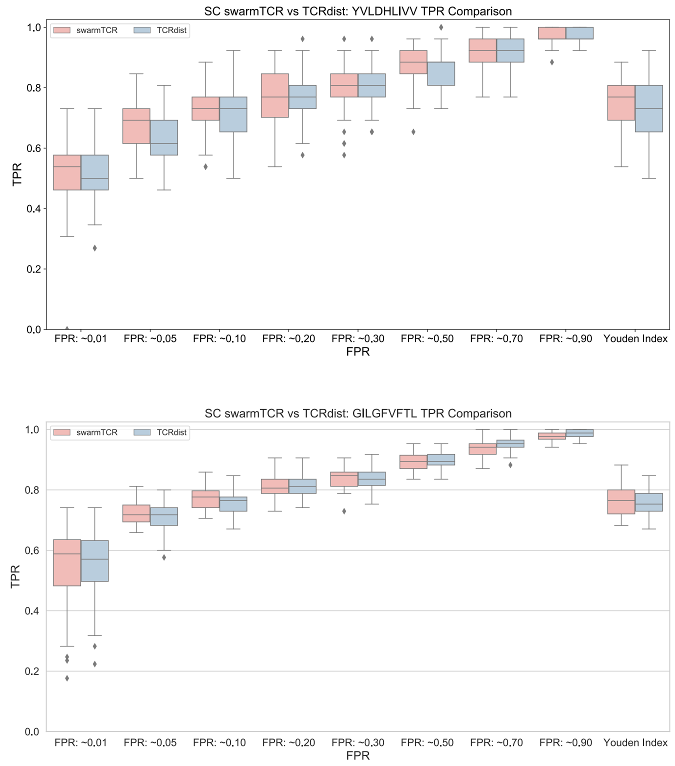

Figure 2: FPRs and their associated TPRs are plotted at thresholds 0.01, 0.1, 0.2, 0.3, 0.5, 0.7, and 0.9 for both swarmTCR and TCRdist (see legend). These plots show the results of the following SC repertoires, top to bottom: YVLDHLIVV, GILGFVFTL.

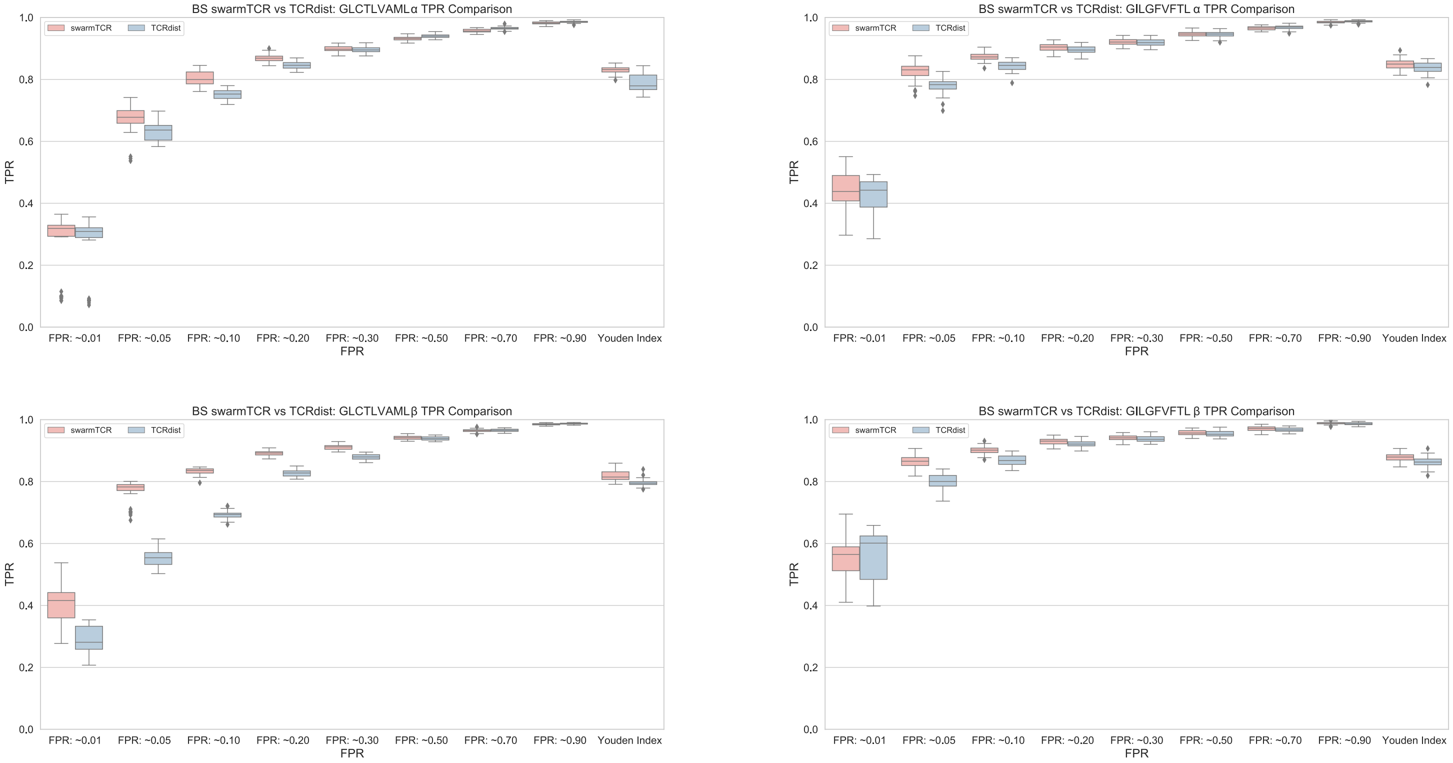

Figure 3: FPRs and their associated TPRs are plotted at thresholds 0.01, 0.1, 0.2, 0.3, 0.5, 0.7, and 0.9 for both swarmTCR and TCRdist (see legend). These plots show the results of the following BS repertoires, top to bottom: GLCTLVAML α, GILGFVFTL α, GLCTLVAML β, GILGFVFTL β.

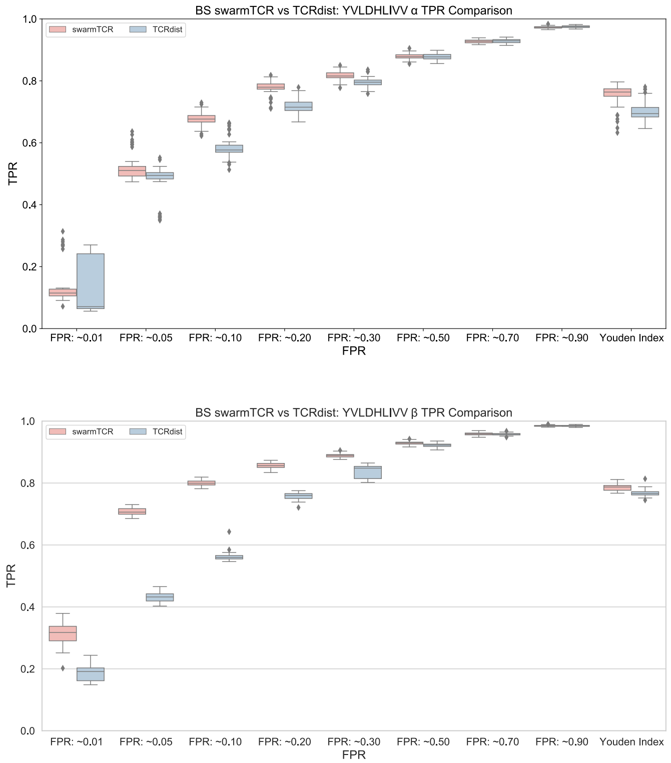

Figure 4: FPRs and their associated TPRs are plotted at thresholds 0.01, 0.1, 0.2, 0.3, 0.5, 0.7, and 0.9 for both swarmTCR and TCRdist (see legend). These plots show the results of the following BS repertoires, top to bottom: YVLDHLIVV α, YVLDHLIVV β.

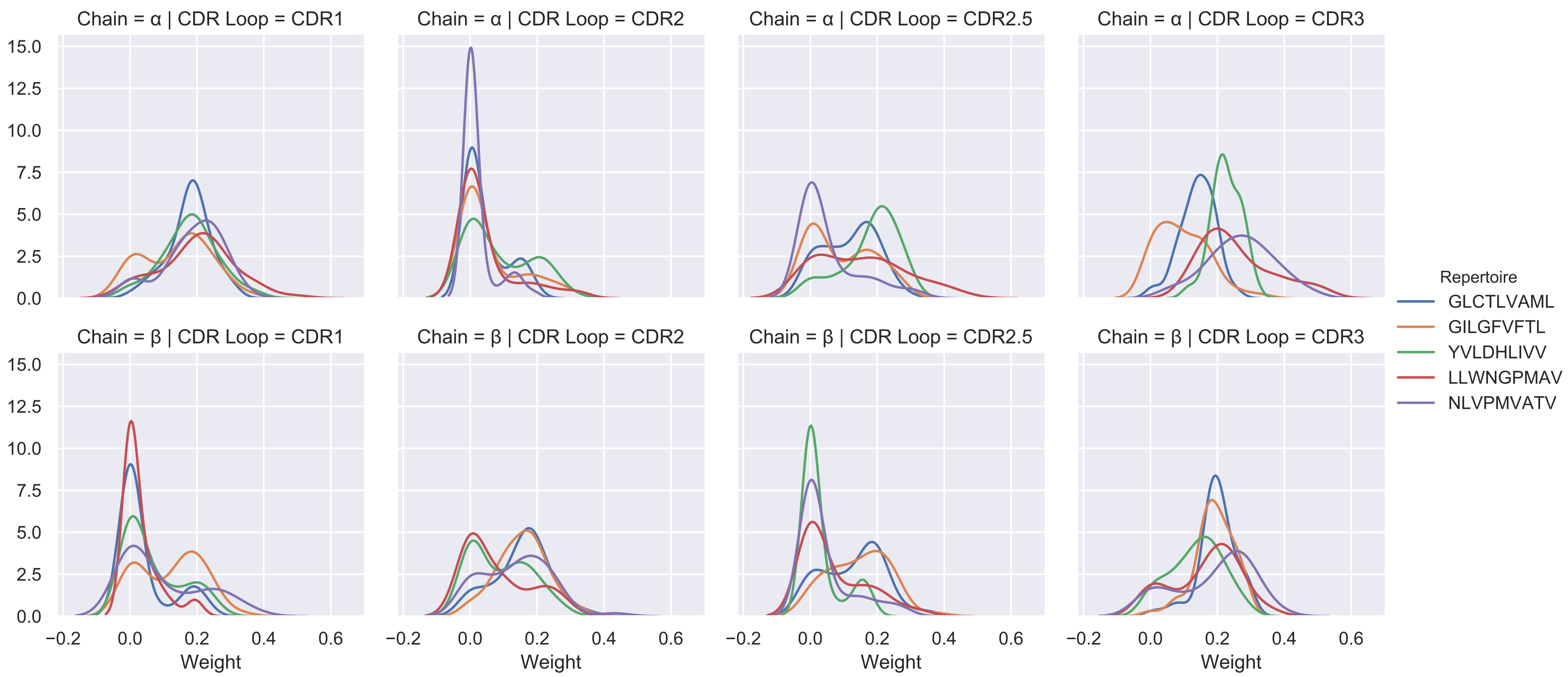

Figure 5: These kernel density plots show the distribution of weights (row 1 = alpha chain, row 2 = beta chain) selected by swarmTCR for the single-cell dataset. Repertoires tested are color-coded (see legend).

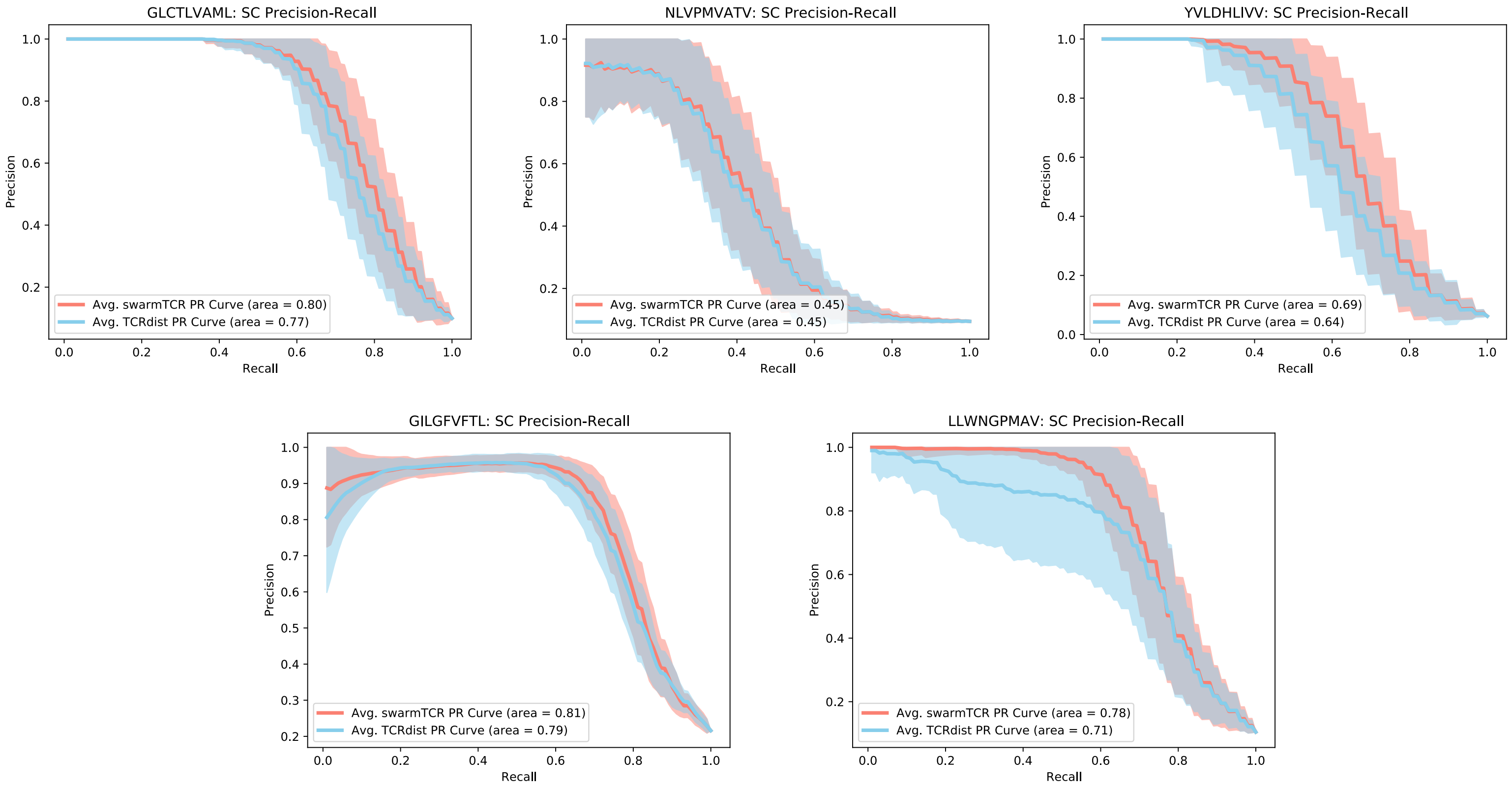

Figure 6: These precision-recall curves show the performance of all tested SC viral repertoires. SwarmTCR and TCRdist on the data used for 50 cross-validation iterations. TCRdist mean curves are in blue and SwarmTCR mean curves are in red, while the shaded regions cover one standard deviation.

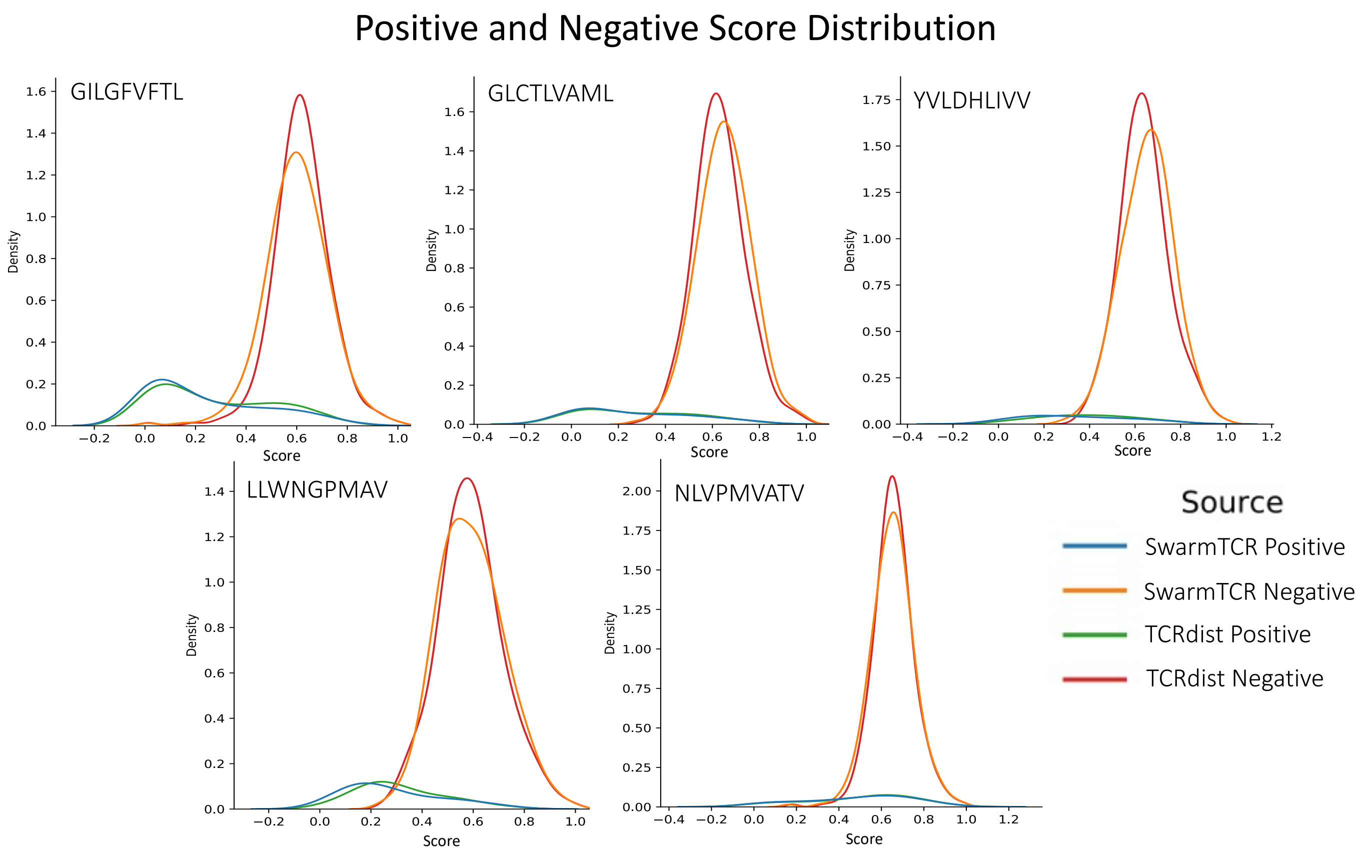

Figure 7: These KDE plots display the average distribution of alignment scores for positive and negative TCRs tested in each repertoire (listed top-left of each plot). The x-axis details alignment score (lower values indicate high similarity) and the y-axis, density.

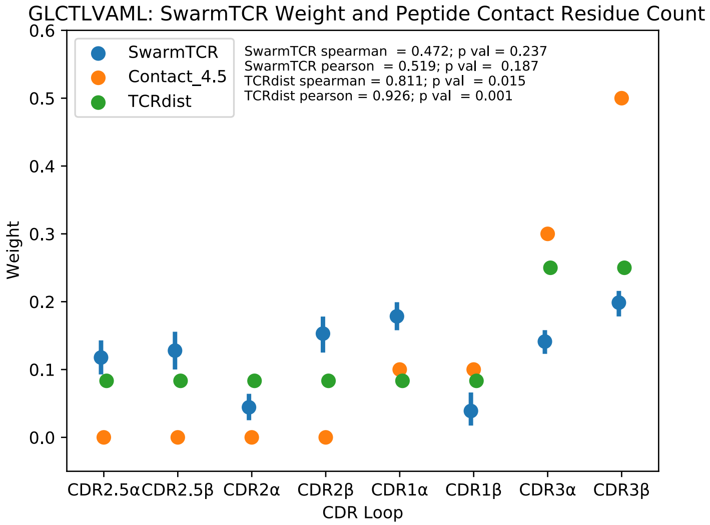

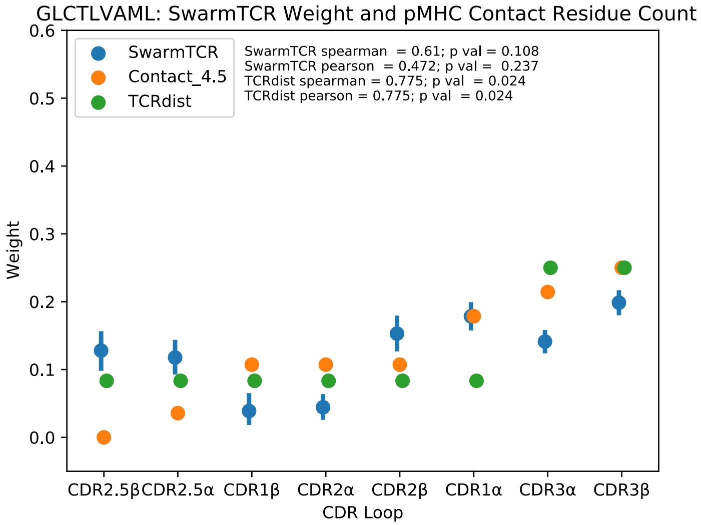

Figure 8: SwarmTCR weights (blue), TCRdist weights (green), and contact residue counts (orange) are plotted in ascending order (contact count) for GLCTLVAML specific TCRs. Peptide and pMHC contact counts are accounted for by the left and right plots, respectively. Spearman and Pearson statistics can be found to the right of the legend.

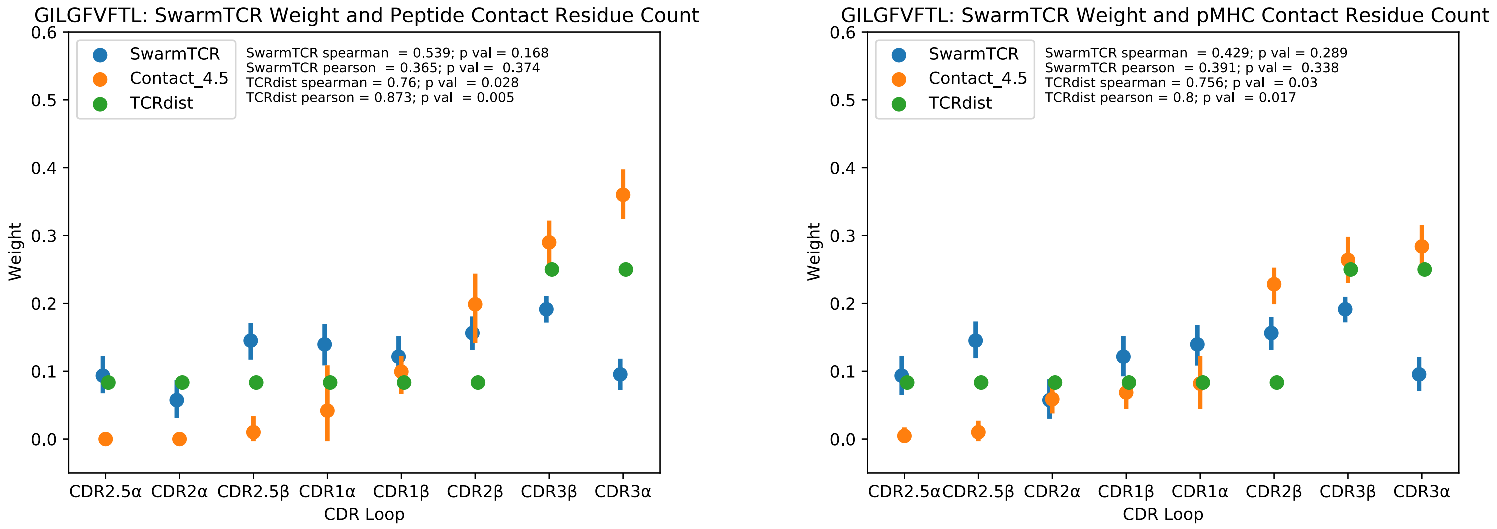

Figure 9: SwarmTCR weights (blue), TCRdist weights (green), and contact residue counts (orange) are plotted in ascending order (contact count) for GILGFVFTL specific TCRs. Peptide and pMHC contact counts are accounted for by the left and right plots, respectively. Spearman and Pearson statistics can be found to the right of the legend.

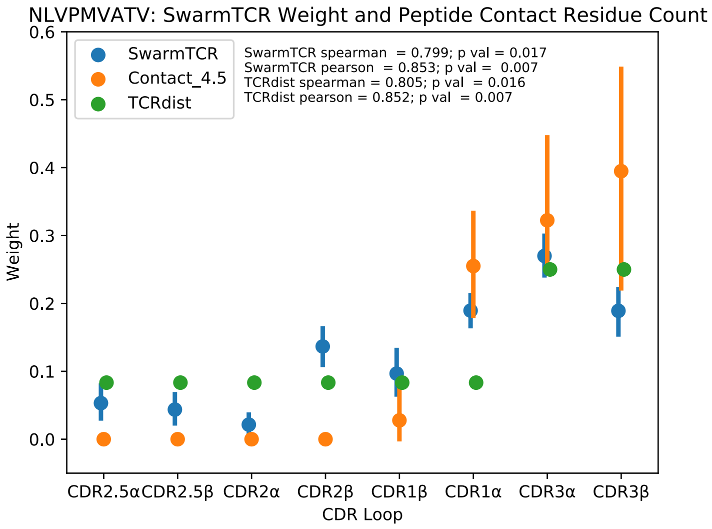

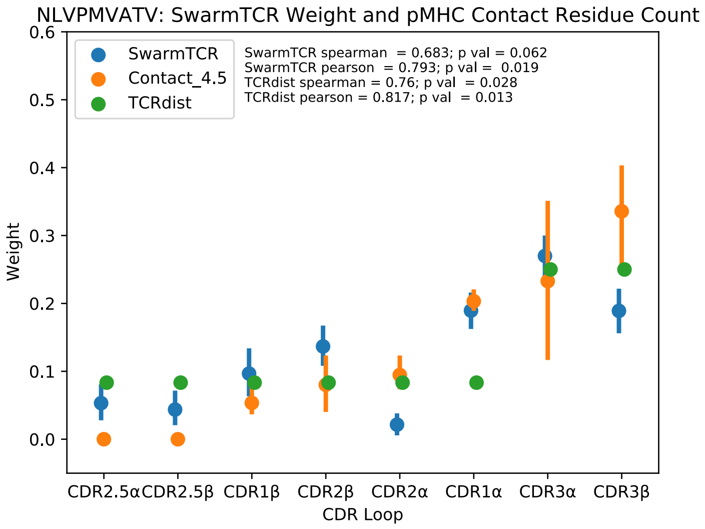

Figure 10: SwarmTCR weights (blue), TCRdist weights (green), and contact residue counts (orange) are plotted in ascending order (contact count) for NLVPMVATV specific TCRs. Peptide and pMHC contact counts are accounted for by the left and right plots, respectively. Spearman and Pearson statistics can be found to the right of the legend.

5EUO	CDR1	CDR2	CDR2.5	CDR3	Total
alpha, pep	0	0	0	3	3
alpha, mhc	1	1	0	5	7
beta, pep	1	2	0	3	6
beta, mhc	1	4	0	4	9
alpha total	1	1	0	8	10
beta total	2	6	0	7	15

1OGA	CDR1	CDR2	CDR2.5	CDR3	Total
alpha, pep	0	0	0	3	3
alpha, mhc	1	2	1	4	8
beta, pep	1	2	0	3	6
beta, mhc	1	4	0	4	9
alpha total	1	2	1	7	11
beta total	2	6	0	7	16

5ISZ	CDR1	CDR2	CDR2.5	CDR3	Total
alpha, pep	1	0	0	4	5
alpha, mhc	4	1	0	3	8
beta, pep	1	2	1	3	7
beta, mhc	2	5	1	5	13
alpha total	5	1	0	7	13
beta total	3	7	2	8	20

2VLJ	CDR1	CDR2	CDR2.5	CDR3	Total
alpha, pep	0	0	0	3	3
alpha, mhc	1	1	1	4	7
beta, pep	1	2	0	3	6
beta, mhc	1	4	0	4	9
alpha total	1	1	1	7	10
beta total	2	6	0	7	16

5JHD	CDR1	CDR2	CDR2.5	CDR3	Total
alpha, pep	0	0	0	5	5
alpha, mhc	3	3	0	6	12
beta, pep	2	2	1	2	7
beta, mhc	2	5	1	4	12
alpha total	3	3	0	11	17
beta total	4	7	2	6	19

2VLK	CDR1	CDR2	CDR2.5	CDR3	Total
alpha, pep	0	0	0	3	3
alpha, mhc	1	1	0	4	6
beta, pep	1	2	0	3	6
beta, mhc	0	4	0	5	9
alpha total	1	1	0	7	9
beta total	1	6	0	8	15

5TEZ	CDR1	CDR2	CDR2.5	CDR3	Total
alpha, pep	2	0	0	2	4
alpha, mhc	3	3	0	2	8
beta, pep	1	0	0	1	2
beta, mhc	1	4	0	4	9
alpha total	5	3	0	4	12
beta total	2	4	0	5	11

2VLR	CDR1	CDR2	CDR2.5	CDR3	Total
alpha, pep	0	0	0	3	3
alpha, mhc	1	1	0	4	6
beta, pep	1	2	0	3	6
beta, mhc	0	4	0	4	8
alpha total	1	1	0	7	9
beta total	1	6	0	7	14

5E6I	CDR1	CDR2	CDR2.5	CDR3	Total
alpha, pep	0	0	0	4	4
alpha, mhc	2	1	1	6	10
beta, pep	1	2	0	2	5
beta, mhc	0	4	0	4	8
alpha total	2	1	1	10	14
beta total	1	6	0	6	13

Figure 11: This table contains CDR loop contact residue data for trimeric TCR/pMHC crystal structures (peptide specificity: GILGFVFTL). Column 1 contains PDB and specifies groups by which contact residue count was determined. Columns 2-6 specify CDR loop or total count for each group described in column 1.

3GSN	CDR1	CDR2	CDR2.5	CDR3	Total
alpha, pep	3	0	0	3	6
alpha, mhc	2	2	0	3	7
beta, pep	1	0	0	4	5
beta, mhc	1	2	0	4	7
alpha total	5	2	0	6	13
beta total	2	2	0	8	12

5D2L	CDR1	CDR2	CDR2.5	CDR3	Total
alpha, pep	3	0	0	4	7
alpha, mhc	3	2	0	3	8
beta, pep	0	1	0	2	3
beta, mhc	0	2	0	4	6
alpha total	6	2	0	7	15
beta total	0	3	0	6	9

5D2N	CDR1	CDR2	CDR2.5	CDR3	Total
alpha, pep	2	0	0	3	5
alpha, mhc	3	2	0	0	5
beta, pep	0	0	0	6	6
beta, mhc	1	3	0	7	11
alpha total	5	2	0	3	10
beta total	1	3	0	13	17

3O4L	CDR1	CDR2	CDR2.5	CDR3	Total
alpha, pep	2	0	0	3	5
alpha, mhc	4	3	0	3	10
beta, pep	1	0	0	5	6
beta, mhc	2	3	0	3	8
alpha total	6	3	0	6	15
beta total	3	3	0	8	14

Figure 12: This table contains CDR loop contact residue data for trimeric TCR/pMHC crystal structures (peptide specificity: NLVPMVATV (green) GLCTLVAML (yellow)). Column 1 contains PDB and specifies groups by which contact residue count was determined. Columns 2-6 specify CDR loop or total count for each group described in column 1.

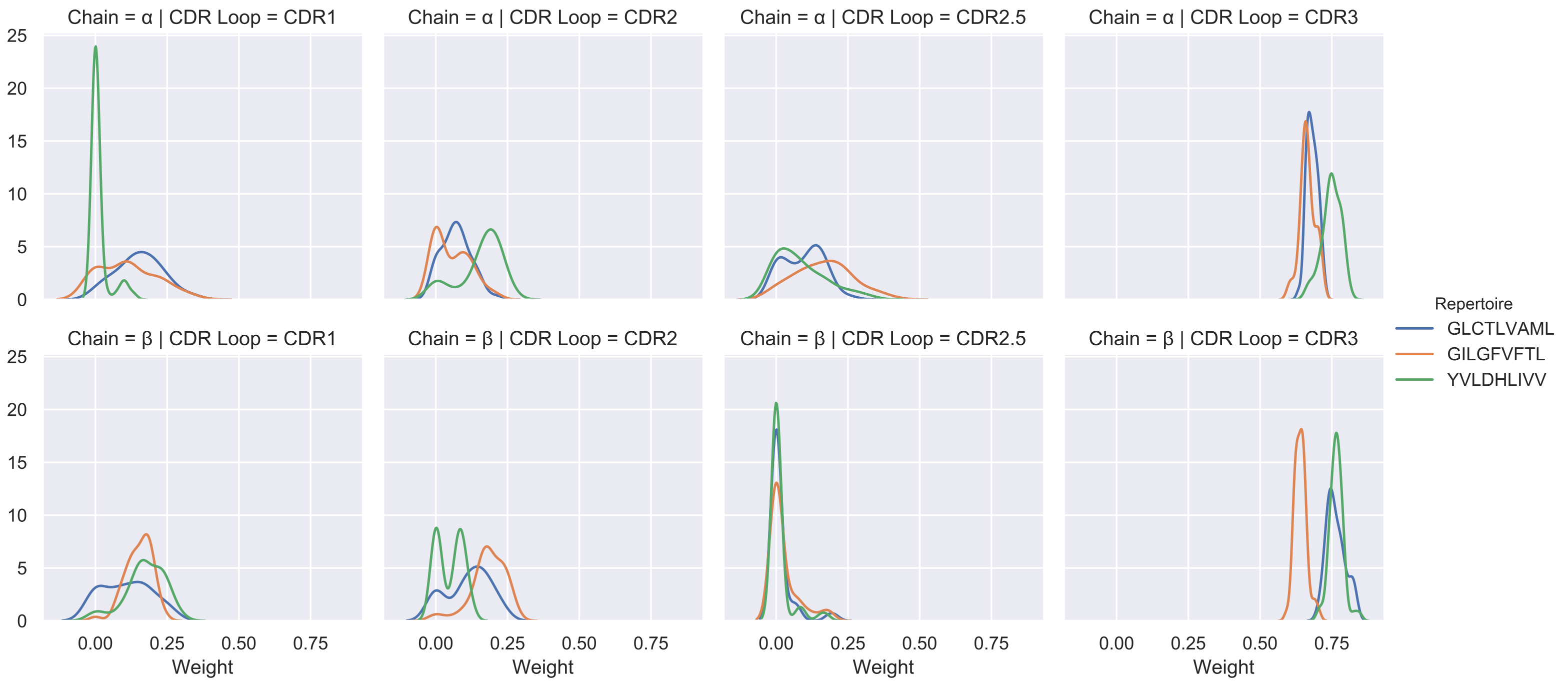

Figure 13: These kernel density plots show the distribution of weights (row 1 = alpha chain, row 2 = beta chain) selected by swarmTCR for the bulk sequencing dataset. Repertoires tested are color-coded (see legend).

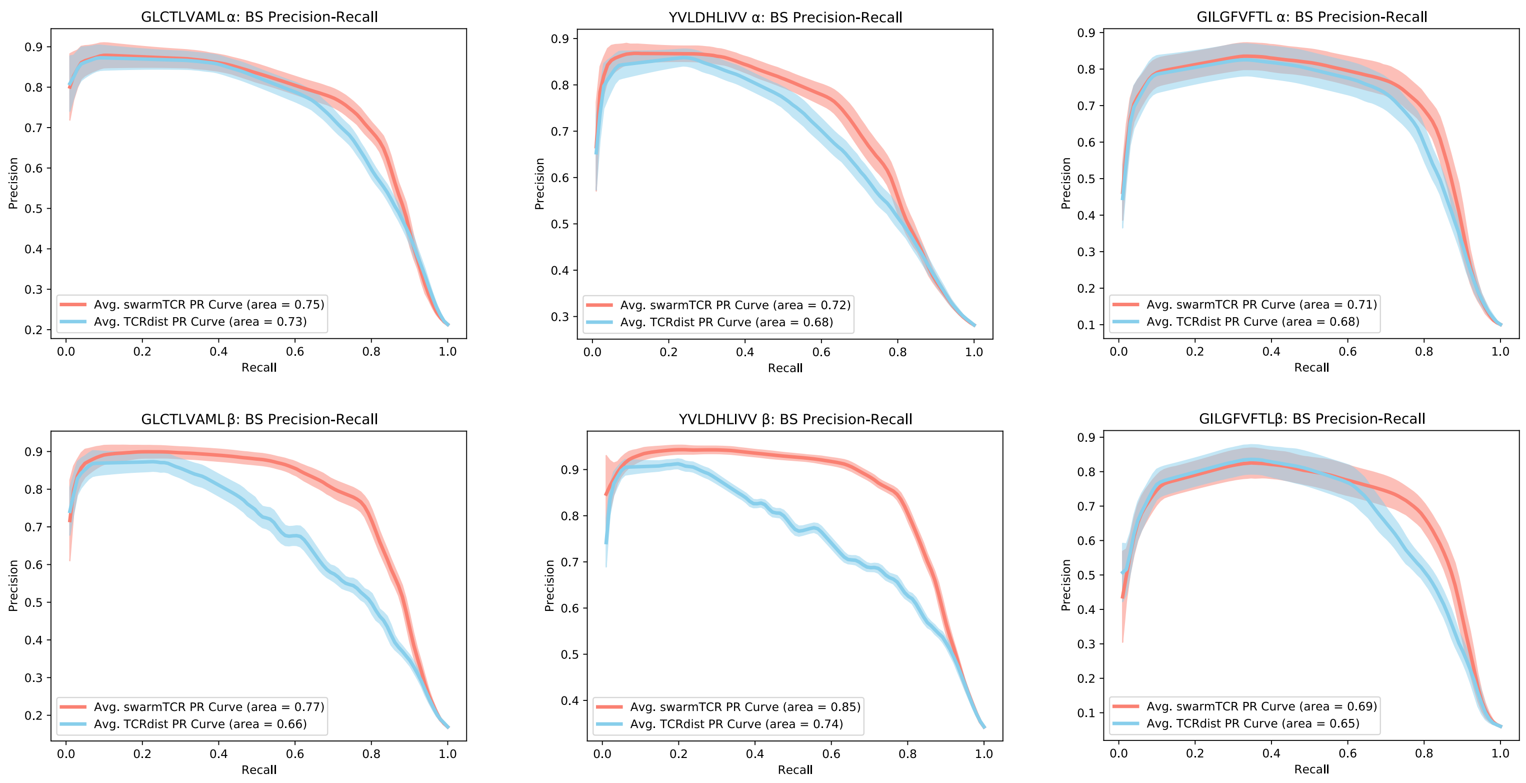

Figure 14: These precision-recall curves show the performance of all tested DS α (top) β (bottom) viral repertoires. SwarmTCR and TCRdist on the data used for 50 cross-validation iterations. TCRdist mean curves are in blue and SwarmTCR mean curves are in red, while the shaded regions cover one standard deviation.
